# Misconceptions Drive COVID-19 Vaccine Hesistancy in Individuals with Inflammatory Bowel Disease

**DOI:** 10.1155/2022/4527844

**Published:** 2022-09-10

**Authors:** Eva Zhang, Arun Gupta, Aysha Al-Ani, Finlay A. Macrae, Rupert W. Leong, Britt Christensen

**Affiliations:** ^1^Dept of Gastroenterology, The Royal Melbourne Hospital Melbourne, Parkville, Australia; ^2^Macquarie University, New South Wales, Sydney, Australia; ^3^Colorectal Medicine and Genetics, The Royal Melbourne Hospital Melbourne, Parkville, Australia; ^4^The University of Melbourne, Melbourne, Australia; ^5^Concord Repatriation General Hospital, Gastroenterology and Liver Services, Concord, Nsw, Aus, Australia

## Abstract

**Background:**

Vaccination is an effective public health measure to combat the SARS-CoV-2 pandemic. However, vaccine “hesitancy” has limited uptake in some, including inflammatory bowel disease (IBD) patients who may have unique concerns influencing uptake.

**Aim:**

The aim of the study is to explore attitudes, concerns, and the influence of different sources of information on COVID-19 vaccine uptake in IBD patients.

**Methods:**

Patients from a specialist IBD clinic at a tertiary hospital in Australia and a national IBD patient society were invited to complete an anonymous online survey regarding COVID-19 vaccination. Demographic characteristics, attitudes towards vaccination, and trust in sources of information were explored. Logistic regression was used to identify variables associated with vaccine uptake.

**Results:**

Of 441 respondents, 93% of respondents had received at least 1 dose of COVID-19 vaccination. Self-perceived risk of being more unwell with COVID-19 infection due to IBD (AOR 5.25, 95% CI 1.96–14.04, *p* < 0.001) was positively associated with vaccine uptake. Concerns regarding the safety of vaccination in pregnancy (OR 0.22, 95% CI 0.08–0.65, *p*=0.006) and of causing an IBD flare (OR 0.28, 95% CI 0.10–0.77, *p*=0.01) were negatively associated with vaccine uptake. In total, 282 (73.7%) responders ranked healthcare workers the most trusted source to obtain information surrounding vaccination.

**Conclusion:**

Vaccine hesitancy in IBD patients is low. Concerns about the safety of vaccination in pregnancy and in causing an IBD flare are both associated with vaccine hesitancy. Healthcare providers play a key role in proactively addressing these misconceptions particularly in the context of emerging virus variants and the availability of boosters.

## 1. Introduction

The SARS-CoV-2 virus was initially detected in December 2019 and is the cause of the syndrome COVID-19. Following its detection, SARS-CoV-2 rapidly spread across the globe and was declared a pandemic by the World Health Organisation in March 2020. COVID-19 has resulted in significant morbidity and mortality [1]. Containment of SARS-CoV-2 has been challenged by the aerosolised nature of transmission, which can be propagated by asymptomatic infected individuals [2]. It was therefore apparent early in the pandemic that vaccination would play an important role in the management strategy of COVID-19.

Vaccine-induced immunity is important in vulnerable cohorts especially those considered immunocompromised. This includes a large proportion of IBD patients who require immune modifying agents as an essential component of their IBD management. Gastrointestinal manifestations of SARS-CoV-2 infection are common as viral entrance to host cells occur via angiotension-converting enzyme 2 (ACE2), which are highly expressed in intestinal cells [5]. Despite initial concern of theoretical risk due to higher expression of ACE2 in inflamed bowel, the SECURE-IBD registry shows IBD patients are not at increased risk of SARS-CoV-2 infection [6].

Numerous SARS-CoV-2 vaccines have now been developed in collaboration with pharmaceutical companies and by state governments. However, IBD patients have been excluded from Phase III “Emergency Use Authorization” approved vaccine trials [5]. This led to initial uncertainty about the influence of immune modifying agents, which theoretically could impact the efficacy of COVID-19 vaccination. Reassuringly emerging studies demonstrate that most IBD patients achieve robust seroconversion rates as measured by antibody response to two doses of COVID-19 vaccines [6]. However, those on high-dose corticosteroids appear less likely to seroconvert with a two-dose schedule [7] and immunity in patients on anti-TNF agents may wane more quickly than in patients on other agents [8].

Whilst uptake of vaccination was initially dependent upon supply issues, globally there have been reports of significant vaccine “hesitancy” hampering current vaccine uptake. Vaccine “hesitancy” has been defined as “delay in acceptance or refusal of vaccination despite availability of vaccination services” [9]. Although vaccination uptake amongst patients with IBD has reportedly been high, there is scope for improvement. [10] Understanding the reasons behind vaccine hesitancy is imperative to overcoming these barriers. This is especially important given evidence of waning immunity after a two-dose schedule of COVID-19 vaccines, and the ongoing threat of new virus variants [11]. Therefore our study aimed to explore the specific aspects of IBD that influenced vaccine hesitancy and more broadly, the perspectives of IBD patients surrounding COVID-19 vaccination. An understanding of the factors that influence the COVID-19 vaccination uptake can be used to tailor education and address misconceptions that may be barriers.

## 2. Methods

An electronic survey was developed following a literature review focusing on vaccine hesitancy. The questionnaire was reviewed prior to finalisation by three senior IBD specialist physicians. The questionnaire was uploaded onto a Research Electronic Data Capture (REDCap) online survey software tool. It comprised of 39 questions in total. Responses to the survey were recorded between 31 October and 16 November 2021. Information sought included baseline demographic data, information regarding the classification, and contemporaneous management of IBD. Respondents were asked about past childhood immunisation, influenza vaccination within the past 12 months, and COVID-19 vaccination. If unvaccinated, respondents were guided to select one or more reasons from a dedicated list of common domains previously identified in literature including: unavailability of vaccines, fear of side effects, concern about rapidity of vaccine development, concern about vaccine efficacy, fear of an IBD flare, and scepticism about the necessity of vaccination. Patients were also asked about the likelihood of future uptake of vaccinations. Questions pertaining to confidence in the safety and efficacy of vaccines, and the safety of vaccination in pregnancy and fertility were presented in the form of a 5-point Likert scale. Respondents were additionally asked to rank the perceived trustworthiness of different information sources about vaccination on a scale of 1 to 7. The survey was approved by the low-risk subcommittee of the Human Research Ethics Committee at Melbourne Health QA2021107.

### 2.1. Recruitment

The survey was sent out without returning identifiable data to the investigators and thus deemed anonymised upon receipt. Patients were recruited from the IBD clinic at The Royal Melbourne Hospital (Victoria, Australia). Additionally, the survey was advertised on the Crohn's & Colitis Australia website, a national patient society for patients with IBD.

### 2.2. Analysis

Descriptive statistics were presented for all respondents. Absolute and relative frequencies were calculated for the categorical variables. Univariate and multivariate logistic regression was used to identify variables associated with vaccine uptake. Variables with a *p* value < 0.10 on univariate analysis were included in multivariate analysis. The crude odds ratio (crude OR) and the adjusted OR (AOR) with 95% confidence intervals (CIs) were calculated. A two-sided *p* value < 0.05 was considered significant. Data were analysed using SPSS (Chicago, IL).

## 3. Results

### 3.1. Patient Characteristics

Demographic and IBD characteristics of the 441 (337 females) respondents are given in Table 1. In total, 262 (59%) had Crohn's disease, 161 (36%) ulcerative colitis (UC), and 18 (4%) inflammatory bowel disease unclassified (IBD-U). In total, 178 (40%) were on 5ASA and 195 (44%) on an immunomodulator. In total, 250 (56%) respondents were on a biologic, with the majority of these on an anti-TNF agent. Most responders were aged between 16 and 59 (86%), whilst 13% were aged 60 years and over. In total, 43% received their IBD care at a public hospital, and 52% with a private gastroenterologist.

### 3.2. Vaccine Uptake

In total, 411 (93%) patients had received at least 1 dose of COVID-19 vaccination. In total, 283 (62%) obtained BNT162b2 Pfizer, 133 (30%) ChAdOx1 nCoV-1 Astra Zeneca, and 5 (1%) mRNA-1273 Moderna. Most agreed that vaccination in general was safe 306 (90%). There were similar rates of confidence in the safety of BNT162b2 (74%), ChAdOx1 nCoV-1 (61%), and mRNA-1273 (62%) vaccines.

In total, 30 (7%) respondents were not vaccinated; 6% of those aged 16–30, 7% of those aged 31–50, and only 1% of 60 + were unvaccinated. Amongst the vaccine hesitant, 5 (16%) were on 5ASA monotherapy and 6(20%) on immunomodulator monotherapy (thiopurine or methotrexate). In total, 12 (40%) were on an anti-TNF agent and amongst this group, 5 were taking concurrent thiopurine or methotrexate. One respondent (3%) was on ustekinumab and 3(10%) on vedolizumab.

Amongst the 30 vaccine hesitant respondents, concern about experiencing an IBD flare with vaccination (*n* = 20) and vaccine safety (*n* = 19) were most commonly identified as reasons for hesitancy. Concerns about how quickly the vaccines were developed (*n* = 17), and scepticism about the efficacy of vaccination (*n* = 6) were also commonly identified. Of the vaccine hesitant, the possibility of getting vaccinated in the future was likely in 3 (11%) and unlikely in 12 (43%). Of note, 13 (45%) were unsure about their future plans for vaccination.

### 3.3. Concerns Pertaining to COVID-19 Pandemic

The main concerns surrounding COVID-19 were fear of getting personally infected (320, 73%), fear of family members being infected (266, 60%), death (144, 33%), long COVID-19 complications (278, 63%), financial implications (70, 16%), employment (68, 15%), unavailability of vaccines (29, 7%), children being unable to go to school (64,15%), prolonged lockdown (119, 27%), and being forced to be vaccinated (42, 10%).

### 3.4. Perspectives about COVID-19 Vaccination and IBD

There were 312 respondents (70%) who agreed/strongly agreed that if they were infected with COVID-19, they would be more unwell due to their IBD. In total, 203 (46%) agreed/strongly agreed that they were concerned that current treatment for IBD reduced vaccine efficacy. In total, 107 (24%) agreed/strongly agreed there was a risk of their IBD flaring with vaccination. Most patients (*n* = 203, 52%) agreed/strongly agreed that vaccination was safe in pregnancy but only 109 (25%) agreed it was safe during breastfeeding. In total, 25 (6%) were concerned it would reduce their fertility.

### 3.5. Factors Associated with Vaccine Uptake

**Figure 1:**
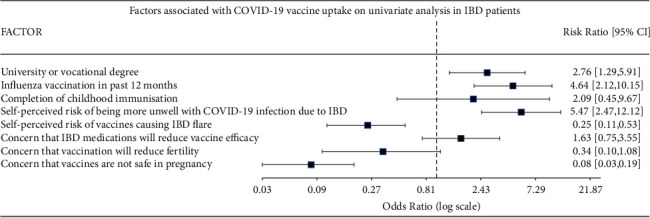
Factors associated with COVID-19 vaccine uptake on univariate analysis in IBD patients.

Multivariate analysis (Table 2) demonstrated past influenza vaccination (OR 3.28, 95% CI 1.34–8.90, *p*=0.009) and self-perceived risk of being more unwell with COVID-19 infection due to IBD was positively associated with COVID-19 vaccine uptake (OR 5.25, 95% CI 1.96–14.04, *p* < 0.001). A higher level of education was positively associated with vaccine uptake on univariate analysis (Figure 1), but this was not significant on multivariate analysis. There was no association with IBD maintenance medication and vaccine uptake including those on biologics or small molecules, combination therapy with anti-TNF and an immune modulator, or those on immune modulator monotherapy.

### 3.6. Factors Associated with Vaccine Hesitancy

The perceived risk of COVID-19 vaccination causing an IBD flare (OR 0.28, 95% CI 0.10–0.77, *p*=0.01) and concern that vaccination is unsafe in pregnancy (OR 0.22, 95% CI 0.08–0.65, *p*=0.006) were both negatively associated with vaccine uptake. There was a trend for those concerned about reduced fertility with vaccination to have increased vaccine hesitancy, but this was not statistically significant.

### 3.7. Trust in Sources of Information

Trust in healthcare workers was high with 282 (74%) responders ranking them the most trusted source to obtain information surrounding vaccination. This was positively associated with vaccination uptake (OR 1.25, 95% CI 1.013–1.54, *p* = 0.03). Trust in information obtained from conventional media (newspaper, television, and radio) (OR 1.74 95% CI 1.16–2.62, *p* < 0.007) and the government (OR 1.75 95% CI 1.32–2.23, *p* < 0.001) was also associated with vaccination uptake. Trust in the Internet was negatively associated with vaccination uptake (OR 0.63 95% CI 0.45–0.87, *p* = 0.006). Social media was ranked the least trusted source of information by 225 (58.6%) but there was no significant association with vaccination uptake. Among groups identified by respondents as helpful in the decision-making process surrounding vaccination, gastroenterologists were the most commonly identified being nominated by 140. Among groups identified by respondents as helpful in the decision-making process surrounding vaccination, 99 nominated their general practitioner, 140 their gastroenterologist, 58 their IBD nurse, and 78 their pharmacist.

## 4. Discussion

This study shows very high rates of confidence in COVID-19 vaccination with 93.2% of respondents having received at least 1 dose. Importantly, of the 3.8% who were unvaccinated, 45% were undecided about future vaccination. Further gastroenterologists were nominated as the most helpful in the decision-making process. This highlights the importance the IBD physician plays in promoting vaccine uptake in our IBD patients.

Vaccine hesitancy stems from a complex interplay of personal and social factors that change with time. Confidence, complacency, and convenience are factors broadly defined by the WHO Working Group surrounding this [9]. Past influenza vaccination was a positive predictor of vaccine uptake and may reflect greater engagement with healthcare and medical recommendations. Confidence in the safety and efficacy of COVID-19 vaccination vaccines has been tempered by the exclusion of IBD patients from Phase 3 vaccine trials. However, concerns about the interaction between vaccination and immunosuppression can be allayed by emerging data demonstrating successful seroconversion across all IBD medication regimens after two doses of mRNA vaccines and ChAdOx1 nCoV-1 [6, 12]. Many IBD patients have additional concerns about their vulnerability to COVID-19 infection. Although the risk of COVID-19 infection in IBD patients is not higher compared to the general population, a self-perceived higher risk of being more unwell with infection due to their IBD was a positive predictor of vaccine uptake. This is in keeping with the notion that complacency about personal risk can influence vaccine hesitancy.

Whilst the COVID-19 vaccine rollout in Australia initially lagged behind Europe and North America, the higher rates of vaccine uptake in this study may reflect increasing confidence over time as the evidence for safety and efficacy rises with global uptake of vaccination. In an Italian survey, 80.3% of IBD patients were willing to be vaccinated [13]. Of the vaccine hesitant 40% answered that their hesitancy was influenced by their IBD. Concerns about the rapidity of vaccine development and the unknown long-term side effects were most commonly selected. In a North American survey, vaccine intent was 60% amongst a general IBD group identified through social media, and 80% amongst a local IBD centre [14]. The hesitant respondents most commonly selected that they would “prefer to see how others tolerate vaccine first” and they were concerned about the long-term safety of vaccines. Both these surveys were conducted in the first two months in which COVID-19 vaccines became available in these countries. In contrast, this survey was conducted approximately six months after COVID-19 vaccination was available to Australians, and almost 12 months post the start of the global rollout [15]. Hence patients here had the benefit of observing the experience in other countries, including those amongst IBD patients. In the state of Victoria in which patients from the IBD clinic were invited to participate, the rate of double-dose vaccination was over 90% for all individuals aged 16 years and over in the period this survey was conducted [16]. The high rate of vaccine uptake in IBD patients in this survey could also reflect the benefit of reassurance from IBD clinicians and nurses. Although healthcare delivery has been challenging in the pandemic, telemedicine has been an effective substitute for most outpatient clinic visits [21]. In an Australian survey of a IBD service surrounding telemedicine, 96% of patients did not alter their immunomodulatory therapy despite over 54% reporting concerns about COVID-19 and their therapy [22]. This reflects the utility of education and reassurance from health professionals throughout the pandemic.

The misconception that vaccination is unsafe in pregnancy is a concerning belief, which was associated with vaccine hesitancy in this survey. The relative rapidity in the development of COVID-19 vaccines and the unknown long-term side effects feature highly amongst IBD patient concerns surrounding vaccine hesitancy [13, 14]. Misconceptions and misinformation from social media sources can fuel these concerns and undermine confidence. Pregnancy or recent pregnancy is a risk factor for severe COVID-19 infection, with associated increased risks of pre-term birth, neonatal intensive care unit admission, and maternal death [19]. Vaccination can reduce the risks of maternal and foetal complications with severe COVID-19 infection in pregnancy [20]. Furthermore, the safety of mRNA and ChAdOx1 nCoV-1 vaccines in pregnancy and breastfeeding has been described [21–23]. There was a trend towards lower vaccine uptake amongst those who were concerned about vaccination and fertility, although this was not statistically significant. There is also no evidence to support an association between vaccination and infertility [24]. Addressing these misconceptions is key in minimising antenatal and perinatal complications with severe COVID-19 infection in IBD patients. All IBD patients, who are planning pregnancy, pregnant, or postpartum, should be encouraged to get vaccinated. General practitioners, gastroenterologists, and obstetricians are well placed to educate patients and should explore this at the clinic or antenatal visits.

The risk of an IBD flare with vaccination was also a prominent concern associated with reduced vaccine uptake. A longitudinal study of 246 IBD patients undergoing mRNA vaccination demonstrated that the frequency of adverse effects, including gastrointestinal symptoms were similar compared to non-IBD patients [25]. Interestingly, individuals on biologic therapy were less likely to experience side effects, particularly after the second dose. Side effects most commonly were fatigue and headache, lasting up to 2 days. Severe side effects were uncommon. These findings are reassuring and should be communicated to IBD patients. The interruption to IBD therapy that may be posed by severe COVID-19 infection and the impacts on IBD disease activity should also be discussed and considered in the decision to vaccinate.

As evidence of waning immunity emerges, policymakers in many countries are recommending “booster” third and fourth vaccination doses for the general population to minimise healthcare and economic consequences [26]. An earlier “third primary dose” and fourth dose for immunocompromised cohorts where a two-dose schedule might not have induced adequate protection has also been recommended [27]. In Australia, IBD patients on high-dose corticosteroids, tofacitinib, and combination immunosuppression are eligible for third primary dose vaccination from 2 months after the second dose in addition to fourth dose [28]. The rapid spread of the more transmissible omicron variant worldwide has led to a more urgent call for boosters. The risk of breakthrough infections is high amongst recipients of two doses of ChAdOx1 nCoV-1 or BNT192 vaccines, as there is a substantial fall in neutralisation titres with omicron [29]. This survey highlights the trust IBD patients place in healthcare professionals, and in particular IBD physicians. As case numbers continue to rise worldwide and omicron displaces delta as the dominant strain, healthcare professionals need to be proactive in engaging patients about COVID-19 vaccination as the need to optimise vaccine uptake becomes more critical.

The limitations of this study include a possible selection bias of younger IBD patients responding to this electronic survey. Recruitment from a national patient society may also select for respondents who are more engaged in their healthcare and medical recommendations. There may be a response bias with those vaccines hesitant more unlikely to respond to the survey. As patients from the IBD clinic invited to respond were from a state with over 50% of national daily new COVID-19 infections, there may be an over-representation of IBD patients who were more motivated to be vaccinated. Respondents to this survey were predominantly female. This may reflect the greater engagement that females have with their healthcare [30, 31]. Similarly, the proportion of female respondents were 75–86% in a North American survey amongst IBD patients [14] Given the survey was anonymous, the number of individuals from whom these respondents were derived cannot be determined.

In conclusion, understanding the motivations behind vaccine uptake and the concerns of the vaccine hesitant is important. As a trusted source of information, healthcare workers are in a prime position to explore and address misconceptions amongst IBD patients. This includes the misconceptions that vaccination will cause an IBD flare, and that vaccination is not safe during pregnancy. This should be a focus of future educational campaigns amongst IBD clinicians, nurses, and patient support groups. Being proactive to encouraging uptake of COVID-19 vaccination including timely primary third dose and booster vaccination will continue to be a priority in order to optimise health outcomes of IBD patients in the pandemic.

## Figures and Tables

**Table 1:** Respondent characteristics.

Survey Items	No. (percentage)
1. IBD diagnosis	
Crohn's disease	262 (59.4%)
Ulcerative colitis	161 (36.5%)
Indeterminate colitis	18 (4.1%)
2. Age group	
16–30	90 (20.4%)
31–59	291 (66.0%)
60+	59 (13.4%)
3. Gender	
Male	98 (22.2%)
Female	337 (76.4%)
4. Location of IBD care	
Public hospital	192 (43.5%)
Private gastroenterologist	225 (51.2%)
General practitioner	23 (5.2%)
5. Highest level of education	
High school	102(23.1%)
TAFE	91 (20.6%)
University	248 (56.2%)
6. Employment status	
Student	27 (6.1%)
Employed	313 (71.0%)
Unemployed	99 (22.4%)
7. Current IBD medications	
Mesalazine or sulfasalazine	178 (40.1%)
Methotrexate	34 (7.7%)
Azathioprine or mercaptopurine	161 (36.5%)
Anti-TNF	159 (36.1%)
Ustekinumab	34 (7.8%)
Vedolizumab	54 (12.2%)
Tofacitinib	3 (0.7%)
Prednisone or oral budesonide	41 (12.3%)
8. Vaccine obtained	
Pfizer BNT162b2	273 (61.9%)
Astra Zeneca ChAdOx1 nCoV-1	133 (30.2%)
Moderna mRNA-1273	5 (1.1%)
9. Factors contributing to vaccine hesitancy amongst those not yet vaccinated	
Unable to schedule vaccine appointment	1 (3%)
Concern about safety of vaccine	19 (63%)
Concern about IBD flaring with vaccination	20 (66%)
Waiting for advice from doctors	4 (13%)
Concern about how quickly vaccines were developed	17 (56%)
Do not believe in efficacy of vaccination	6 (20%)

**Table 2:** Factors associated with vaccine uptake.

Factor	Univariate OR (95% confidence interval)	Multivariate OR (95% confidence interval)
Male sex	0.71 (0.30 – 1.65) *P*=0.62	
University or vocational degree	2.76 (1.29 – 5.91), *p*=0.009	2.03 (0.77 – 5.29), *P*=0.14
Crohn's disease	1.24 (0.54 – 2.84), *P*=0.60	
Current biologic or small molecule	1.09 (0.52 – 2.29), *P*=0.86	
Current thiopurine or methotrexate	1.04 (0.49 – 2.19), *P*=0.92	
Combination anti-TNF and immunomodulator use	0.75 (0.30 – 1.91), *P*=0.54	
Influenza vaccination in past 12 months	4.64 (2.12 – 10.15) *p* < 0.001	3.28 (1.34 – 8.9), *p*=0.009^*∗*^
Completion of childhood immunisation	2.09 (0.45 – 9.67), *P*=0.34	
Confidence in safety of vaccines in general	6.87 (2.90 – 16.26), *p* < 0.001	2.16 (0.73 – 6.37), *P*=0.162
Self-perceived risk of being more unwell with COVID-19 infection due to IBD	5.47 (2.47 – 12.12) *p* < 0.001	5.25 (1.96 – 14.04), *p* < 0.001^*∗*^
Self-perceived risk of vaccines causing IBD flare	0.25 (0.11 – 0.53), *p* < 0.001	0.28 (0.0.10 – 0.77), *p*=0.01^*∗*^
Concern that IBD medications will reduce vaccine efficacy	1.63 (0.75 – 3.55), *P*=0.21	
Concern that vaccination will reduce fertility	0.34 (0.10 – 1.08), *p*=0.06	0.57 (0.13 – 1.00), *P*=0.44
Concern that vaccines are not safe in pregnancy	0.08(0.03 – 0.19), *p* < 0.001	0.22 (0.8 – 0.65), *p*=0.006^*∗*^

^*∗*^Denotes significance *P*=0.05.

## Data Availability

The data that support the findings of this study are available from the corresponding author EZ, upon request.
